# Differences of tensile strength in knot tying technique between orthopaedic surgical instructors and trainees

**DOI:** 10.1186/s12893-021-01079-5

**Published:** 2021-02-06

**Authors:** Kengo Harato, Mitsuru Yagi, Kazuya Kaneda, Yu Iwama, Akihiko Masuda, Yosuke Kaneko, Akihito Oya, Noboru Matsumura, Taku Suzuki, Robert Nakayama, Shu Kobayashi

**Affiliations:** 1 Department of Orthopedic Surgery, Keio University School of Medicine, 35 Shinanomachi, Shinjukuku, Tokyo, 160-8582 Japan; 2 Keio Orthopedic Advancing Squad for the Interactive Study (OASIS), Keio University School of Medicine, Tokyo, Japan

**Keywords:** Knot-tying, Tensile strength, Instructor, Trainee, Knot slippage

## Abstract

**Background:**

Knot tying technique is an extremely important basic skill for all surgeons. Clinically, knot slippage or suture breakage will lead to wound complications. Although some previous studies described the knot-tying technique of medical students or trainees, little information had been reported on the knot-tying technique of instructors. The objective of the preset study was to assess surgeons’ manual knot tying techniques and to investigate the differences of tensile strength in knot tying technique between surgical instructors and trainees.

**Methods:**

A total of 48 orthopaedic surgeons (postgraduate year: PGY 2–18) participated. Surgeons were requested to tie surgical knots manually using same suture material. They were divided into two groups based on each career; instructors and trainees. Although four open conventional knots with four throws were chosen and done with self-selected methods, knot tying practice to have the appropriate square knots was done as education only for trainees before the actual trial. The knots were placed over a 30 cm long custom made smooth polished surface with two cylindrical rods. All knots were tested for tensile strength using a tensiometer. The surgical loops were loaded until the knot slipped or the suture broke. The tensile strength of each individual knot was defined as the force (N) required to result in knot failure. Simultaneously, knot failure was evaluated based on knot slippage or suture rupture. In terms of tensile strength or knot failure, statistical comparison was performed between groups using two-tailed Mann–Whitney U test or Fisher exact probability test, respectively.

**Results:**

Twenty-four instructors (PGY6–PGY18) and 24 trainees (PGY2–PGY5) were enrolled. Tensile strength was significantly greater in trainees (83.0 ± 27.7 N) than in instructors (49.9 ± 34.4 N, P = 0.0246). The ratio of slippage was significantly larger in instructors than in trainees (P < 0.001). Knot slippage (31.8 ± 17.7 N) was significantly worse than suture rupture (89.9 ± 22.2 N, P < 0.001) in tensile strength.

**Conclusions:**

Mean tensile strength of knots done by trainees after practice was judged to be greater than that done by instructors in the present study. Clinically, knot slippage can lead to wound dehiscence, compared to suture rupture.

## Background

Knot-tying technique is an extremely important basic skill for all orthopaedic surgeons [1–3]. Clinically, knot slippage or breakage will lead to wound complication such as dehiscence. For instance, in terms of knee replacement surgery, the suture tension in the proximal capsule showed a marked increase along with an increase in the knee flexion angle, and the maximum tension was 44 N based on a previous study [4]. However, knot strength varied widely (16–328 N) among expert surgeons [2]. Therefore, security of knot-tying is essential for all surgeries. According to previous studies, it has been a technically demanding task to obtain an appropriate security of knot-tying especially for young surgeons, as approximately 45% of trainees were unable to tie knots securely and about 15% of knots were judged as dangerous [5]. On the other hand, knot security had poor correlation with surgical experience and more important correlation with the differences in knot tying technique and type of knot created [6]. As a clinical education for trainees, instructors usually teach the way to perform an appropriate knot tying technique in the surgical field. However, no instructors really know how appropriate their own knots. Although some previous studies described the knot-tying technique of medical students or trainees after practice [1, 3, 5, 7, 8], little information had been reported on the difference of knot-tying technique between surgical instructors and trainees.

The main purpose of the present study was to assess surgeons’ manual knot tying techniques and to investigate the differences of tensile strength in knot-tying technique between surgical instructors and trainees. The second aim was to let surgical instructors and trainees know the accuracy of their own knots. It was hypothesized that knot strength would vary among all surgeons including surgical instructors and trainees, and there were no differences of knot-tying technique between surgical instructors and trained trainees.

## Methods

**Fig. 1:**
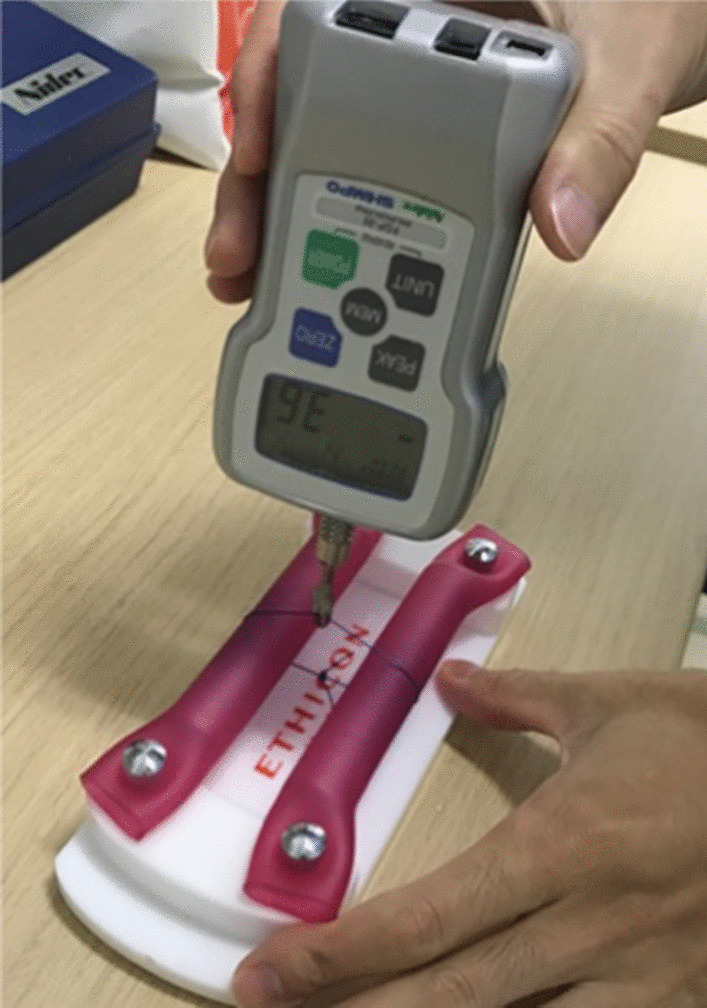
Tensile strength was measured using a portable tensiometer

A total of 48 surgeons (9 females and 39 males) participated in the present study. From May to July in 2019 and May to July in 2020, surgical instructors and trainees working at department of orthopaedic surgery in our hospital were invited to participate in the present study. Average career lifetime as a doctor was 6.7 years (postgraduate year: PGY 2–18). Surgeons with career lifetime greater than 20 years were excluded from the current study, as most of authors had career lifetime with longer than 20 years. Attending surgeons were provided with consent confirming their voluntary participation. Surgeons were divided into two groups based on each career; instructors and trainees. They were requested to tie surgical knots manually using same suture material including No. 1 PDS (Ethicon, Inc, Somerville, New Jersey). Four open conventional knots with four throws were chosen for the present investigation and done with self-selected methods for each surgeon. However, before the actual trial, knot tying practice to have the appropriate square knots was done as education only for trainees using textbook and/or string. The knots were placed over a 30 cm long custom made smooth polished surface with two cylindrical rods (Ethicon, Inc, Somerville, New Jersey) (Fig. 1). The rod was anchored firmly on both ends during knot tying. All knots were tested for tensile strength using a tensiometer (Digital force gauge, Nidec cooperation, Kyoto, Japan). The surgical loops of No.1 PDS were loaded until the knot either broke or slipped. The tensile strength of each individual knot was defined as the force (N) required to result in knot failure by slippage or rupture. Simultaneously, knot failure was evaluated based on knot slippage or suture rupture. This educational study was done using a verbal consent, which was approved by the Ethics Committee and Institutional Review Board of our university hospital (IRB No. 20190174). In our hospital, a written consent was not applicable regarding the educational study as any patients were not involved.

In terms of tensile strength or knot failure, statistical comparison was performed between groups using two-tailed Mann–Whitney U test or Fisher exact probability test, respectively. All statistical analyses were done with the Microsoft Excel Statistical Package, version 2019 (Social Survey Research Information, Tokyo, Japan). Moreover, tensile strength was compared between suture rupture and knot slippage using two-tailed Mann–Whitney U test. In terms of gender difference, tensile strength was compared using two-tailed Mann–Whitney U test. Furthermore, Pearson's coefficient was used to analyze correlations between postgraduate year and tensile strength. The threshold for statistical significance was set at a P value of < 0.05.

## Results

**Fig. 2:**
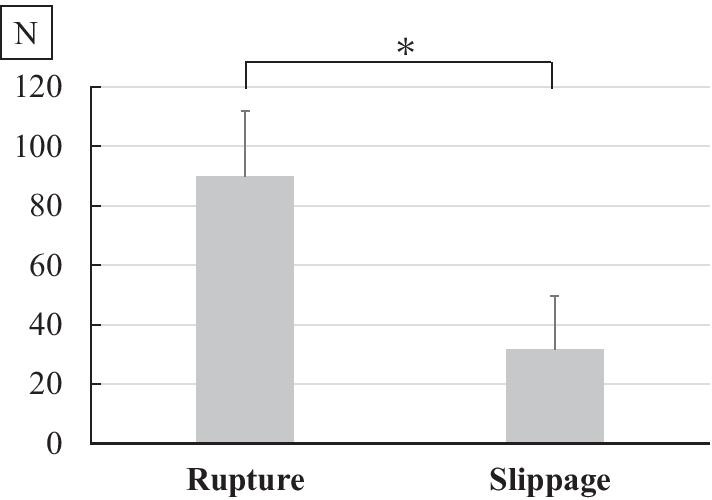
Tensile strengths of suture rupture and knot slippage

**Fig. 3:**
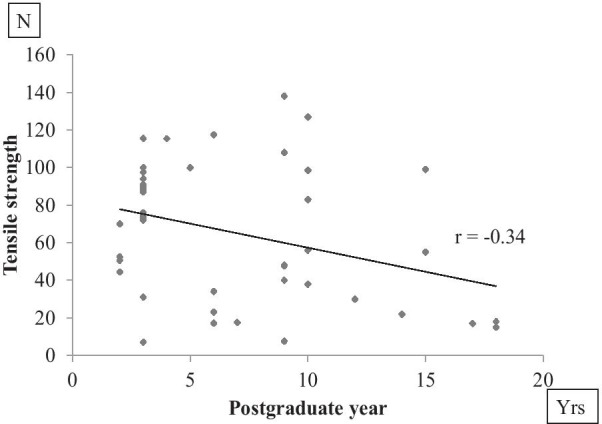
Relationship between postgraduate year and tensile strength

A total of 24 instructors (PGY6–18) and 24 trainees (PGY2–5) were enrolled in the present study. Subspecialties for instructors were spine (3 surgeons), tumor (2 surgeons), upper extremity (9 surgeons), and lower extremity (10 surgeons), whereas subspecialty was not available for trainees. Average careers as a doctor were 10.6 ± 3.7 years for instructors and 3.0 ± 0.6 years for trainees since they graduated from medical school or university (Table 1). Two females and 22 males were allocated in instructors, whereas 7 females and 17 males were in trainees. Mean tensile strengths were 77.3 ± 25.6 (N) for trainees and 54.0 ± 40.1 (N) for instructors. Knot strength varied widely (7–138 N) among surgeons. Interestingly, mean tensile strength was significantly greater in trainees than in instructors (P = 0.0246). Knot slippage was observed in 17 out of 24 instructors and 3 out of 24 trainees (Table 1). Similarly, the ratio of slippage was significantly larger in instructors than in trainees (P < 0.001). Concerning the knot failure, tensile strengths of suture rupture and knot slippage were 89.9 ± 22.2 (N) and 31.8 ± 17.7 (N), respectively. Knot slippage was significantly worse than suture rupture as to tensile strength (P < 0.001) (Fig. 2). Knot strengths in females and males were 71.4 ± 30.8 (N) and 64.1 ± 36.6 (N), respectively. There was not significantly different between female and male surgeons (P = 0.59). Tensile strength was negatively correlated with postgraduate year of surgeons (r = − 0.34; 95% confidence interval, − 0.56 to − 0.06, P = 0.02) (Fig. 3).

**Table 1:** Comparison of data between instructors and trainees (mean ± S.D.)

	Instructors	Trainees	P value^a^
Career (years)	10.6 ± 3.7	3.0 ± 0.6	< 0.001
Female/male	2/22	7/17	0.136
Tensile strength (N)	54.0 ± 40.1	77.3 ± 25.6	0.0246
Rupture/slippage	7/17	21/3	< 0.001

^a^Values obtained using two-tailed Mann–Whitney U test or Fisher exact probability test

## Discussion

The result of the present study supported our hypothesis that knot strength would vary among all surgeons including surgical instructors and trainees (7–138 N). The main finding of the present study was that knot-tying technique used by trainees after practice was judged to be superior to those used by instructors.

Batra et al. assessed the influence of surgeon's tying technique on knot security using 0 and 2–0 monofilament and multifilament nylon sutures with a portable tensiometer, and concluded that knot-tying technique used by medical students was judged to be superior to those used by surgeons after students had practice knot-tying to make four-throw square knots appropriately [7]. Similarly, from the present study, mean tensile strength was significantly greater in trainees than in instructors. Therefore, practice seemed to be essential both for trainees and instructors. In addition, trainees were usually taught regarding knot-tying technique during the actual surgery at the operating room and thus tended to perform the knot-tying carefully based on the instruction from the senior surgeons [6]. On the other hand, surgical instructors were confident with knot-tying technique and less prudent to make knots in the present simulation as they had rarely experienced major wound complications during the surgical procedures so far, while the true reason that some instructors could not make the appropriate knots was unknown. However, in this trial, three trainees could not make the secure knots even if the practice was done. This seemed to be a problem, and thus the education program should be continuous [9]. Ind et al. indicated that knot-tying workshops could improve the ability of trainees to tie reef knots [5]. In terms of knot strength, a previous cadaveric study suggested that the suture tension in the proximal capsule at the knee joint showed a marked increase along with an increase in the knee flexion angle and the maximum tension was 44 N [4]. Therefore, knot slippage can be risky for capsule dehiscence, as tensile strengths for slippage was 31.8 ± 17.7 (N) and the incidence of dangerous knots was high in the present study even if No. 1 PDS was used. Appropriate knot-tying method to avoid wound dehiscence is a fundamental technique even for surgical instructors as well as trainees.

Several limitations should be described. First, knot-tying methods could not be standardized in the present study, even if four-throw square knots were recommended for all surgeons. Therefore, it was unknown whether weaker knots were square or not. Second, it is unknown whether knot slippage will lead to the actual clinical complication such as postoperative bleeding or wound dehiscence. Third, only monofilament material including No. 1 PDS was used in the current investigation. Thus, the result may be different if a braided material is utilized. Lastly, although sample size calculation was done based on the initial 20 samples (10 in each group), sufficient power was not obtained as standard deviation was large in each group even if significant differences were obtained using data from 48 surgeons. However, the results of the present study offer useful information when considering the accuracy of knot-tying technique both in instructors and trained trainees.

## Conclusion

Knot-tying technique used by trainees after practice was judged to be superior to those used by instructors in the present study. Clinically, knot slippage can be risky for wound dehiscence, compared to knot breakage.

## Data Availability

The datasets used and/or analysed during the current study available from the corresponding author on reasonable request.

## Abbreviation

PGY: Postgraduate year
